# Six-Axis Robotic Milling for Enhancing Surface Quality and Dimensional Accuracy of Fused Granular Fabrication Parts

**DOI:** 10.3390/polym18050608

**Published:** 2026-02-28

**Authors:** Rui Zhang, Xiping Li, Youqiang Yao, Sisi Wang, Yu Zhou, Zhonglue Hu

**Affiliations:** College of Engineering, Zhejiang Normal University, Jinhua 321004, China; zhangrui09190@163.com (R.Z.); yaoyouqiang@zjnu.edu.cn (Y.Y.); sisi.wang@zjnu.edu.cn (S.W.); zhouyu112910@163.com (Y.Z.)

**Keywords:** fused granular fabrication, PP-GF composites, milling parameters, robotic arm milling, multi-objective optimization, surface quality

## Abstract

Fused granular fabrication (FGF) offers high deposition efficiency and low material cost for large-scale mold production, but commonly yields parts with surface defects and dimensional deviations. This study develops a six-axis robotic post-processing workstation that integrates multi-DOF toolpath planning and real-time communication to flexibly machine FGF components with complex geometries. Using short-fiber-reinforced polypropylene (PP-GF), robotic milling experiments were performed, and spindle speed, feed rate, and cutting depth were systematically optimized to enhance surface quality and dimensional accuracy. The NSGA-III algorithm optimizes parameters, thereby increasing machining efficiency by 4.9% and reducing surface roughness by 12.35%. Results show that the proposed platform effectively improves the machining performance of FGF-printed parts, demonstrating its feasibility for high-precision post-processing. The work provides a practical technical route for the hybrid additive–subtractive manufacturing of large 3D-printed structures.

## 1. Introduction

The accelerated growth of aerospace, energy equipment, and other engineering transportation industries has led to an increasing demand for large-scale and complex structural components, thereby imposing stringent requirements on manufacturing efficiency, dimensional accuracy, and cost-effectiveness [1]. Conventional subtractive manufacturing processes exhibit inherent limitations in manufacturing efficiency and material utilization, particularly for large-scale and complex geometries. In contrast, additive manufacturing (AM) has emerged as an effective approach for fabricating large-scale components due to its design flexibility and reduced material waste [2]. Among extrusion-based AM technologies, fused granular fabrication (FGF) has garnered considerable attention as a promising method for large-scale production, owing to its high deposition efficiency and low raw material cost [3,4,5]. FGF enables the direct use of thermoplastic pellets, showing strong potential for manufacturing large-scale polymer and fiber-reinforced composite structures [6]. Existing studies on FGF have mainly focused on process development, material extrusion behavior, and mechanical performance of as-printed parts [7,8]. However, the surface quality and dimensional accuracy of FGF-fabricated components remain critical challenges, particularly for applications with high precision requirements.

However, the pursuit of high deposition efficiency in FGF is accompanied by a critical limitation, namely insufficient surface resolution, commonly referred to as the efficiency–resolution trade-off [9]. During the deposition process, the staircase effect and uneven interlayer bonding lead to increased surface roughness, making it difficult for as-built parts to meet stringent engineering requirements such as assembly precision, aerodynamic performance, or service life [10,11]. Consequently, post-processing is generally required to improve the surface quality and geometric accuracy of FGF components. Common post-processing methodologies include milling, grinding, and polishing [12,13,14]. Among these methods, milling is recognized as particularly effective due to its high material removal rate and superior machining accuracy [15,16]. A multitude of studies have demonstrated that the milling process of AM components can lead to a substantial reduction in surface roughness and dimensional deviations, thereby expanding the range of their engineering applications [17,18]. However, for large-scale and complex-shaped AM components, the workspace and flexibility limitations of conventional CNC machine tools necessitate the use of robotic milling solutions.

A comparison of conventional CNC machine tools and six-degree-of-freedom (6-DOF) robotic arms reveals the former’s distinct disadvantages in processing large-scale and complex-shaped parts [19]. Robotic systems offer two major advantages in this regard. Firstly, they provide a larger workspace. Secondly, they enhance flexibility in posture adjustment [20]. This makes them particularly suitable for machining freeform surfaces [21]. In recent years, robotic arms have seen increased application in the post-processing of AM components, including milling and finishing of metals and composites [22,23,24]. Nevertheless, studies specifically addressing the robotic milling of FGF-fabricated components, particularly fiber-reinforced thermoplastics, remain limited, and several critical issues have yet to be systematically resolved. First, the material removal mechanisms of FGF-produced, fiber-reinforced thermoplastics differ fundamentally from those of conventional metals, and their machinability remains insufficiently understood [25,26,27]. Secondly, robotic arms, due to their limited stiffness, are susceptible to vibration during milling, which compromises machining accuracy and process stability [28]. Furthermore, in the context of large-scale workpieces, the efficient planning of toolpaths and the optimization of process parameters to balance machining efficiency and surface quality remains a challenging problem [29].

To address the above knowledge gaps, this study systematically investigates the robotic milling behavior of large-scale short glass fiber-reinforced polypropylene (PP-GF) components fabricated via fused granular fabrication (FGF). The originality of this work lies in three key aspects. First, a dedicated robotic post-processing platform for FGF-fabricated PP-GF components is developed, enabling the integrated measurement of cutting forces, milling temperature, and surface roughness. Second, the individual and combined effects of spindle speed, feed rate, and cutting depth on machining performance are quantitatively analyzed using orthogonal experiments and statistical methods, providing process-level insights specific to FGF-based composites [30,31,32]. Third, a multi-objective optimization framework combining regression modeling and the NSGA-III algorithm is proposed to balance cutting force, surface quality, and machining efficiency in robotic milling [33]. The results of this study not only deepen the understanding of the machinability of FGF-fabricated, fiber-reinforced polymers but also provide practical guidance for the high-precision post-processing of large-scale AM mold components.

## 2. Materials and Methods

### 2.1. Materials

The material selected for this study is short glass fiber-reinforced polypropylene (PP-GF). The glass fiber content is 30 wt.%. The detailed physical and mechanical properties of the PP-GF are listed in Table 1.

### 2.2. The Preparation of FGF-Printed Specimen

**Figure 1:**
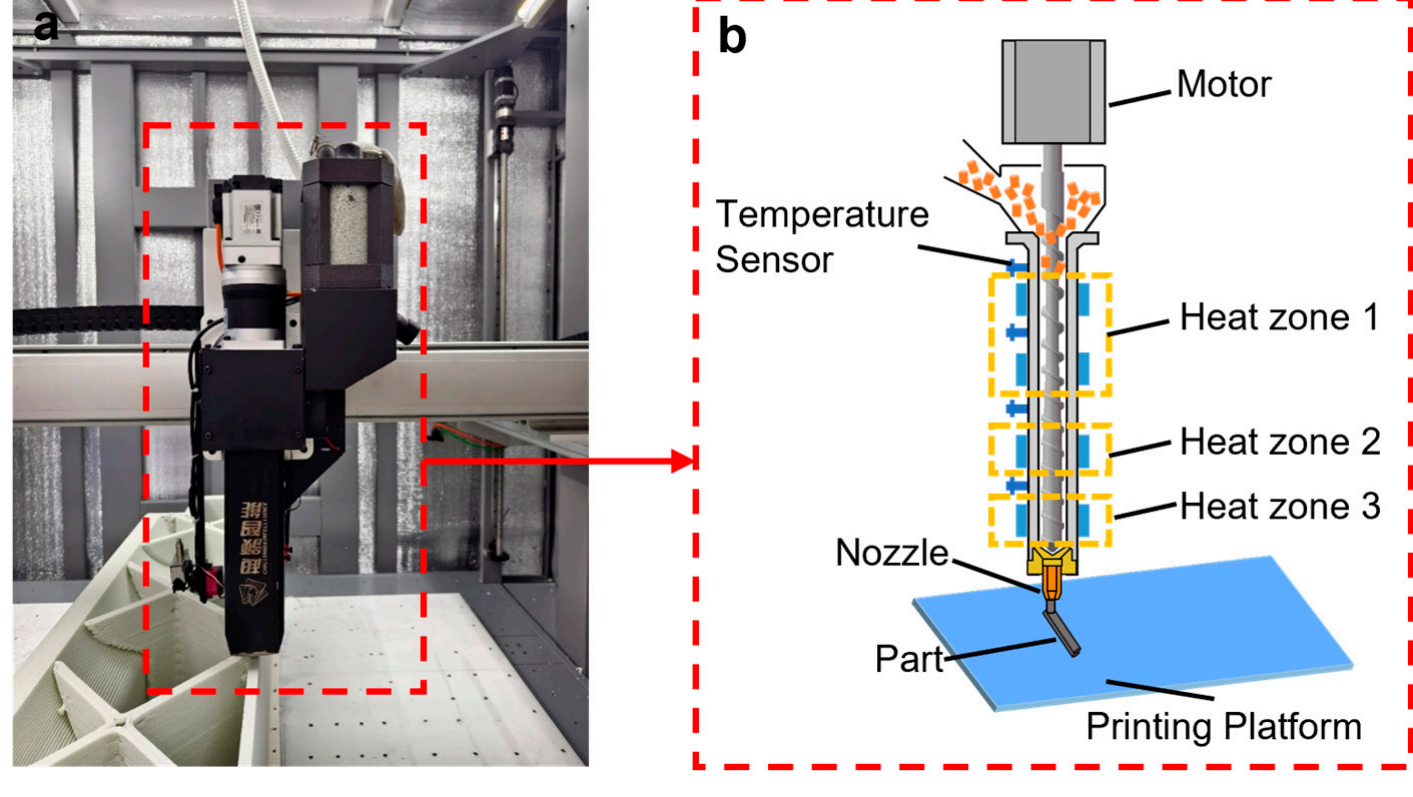
The FGF 3D-printer used for this study: (**a**) picture of the FGF printer; (**b**) schematics of the screw-extruder.

In this study, the 3D-printed specimens were fabricated with a custom-designed, high-throughput FGF 3D printer (Uni-Print 1500, Zhejiang Chaoling Inc., Jinhua, China), as shown in Figure 1a. The key component of this printer is the single-screw extruder, which is equipped with four independent heat segments and a 750 W servo motor(Zhejiang HCFA Technology Co., Ltd., Longyou, China). The pelletized feedstock was fed directly into the 20 mm-diameter screw before subsequently extruded through the 4.0 mm-diameter nozzle, as shown schematically in Figure 1b. The key printing parameters, including the temperature, line width, and infill density, are listed in Table 2.

### 2.3. The Robotic Arm-Based Milling Platform

**Figure 2:**
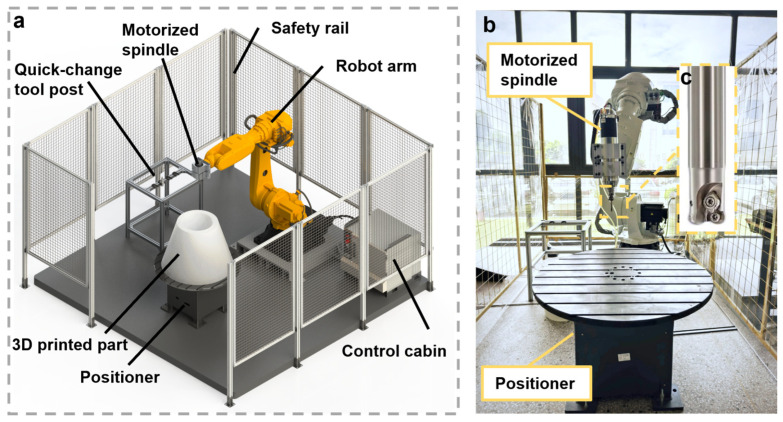
Schematics of the six-axis robotic-arm milling workstation. (**a**) Schematics of the robotic arm-based milling station, which consists of the robot arm, spindle, positional, and the parts to be processed, (**b**) pictures of the actual milling workstation, and (**c**) the magnified image of the endmill.

Figure 2a illustrates the assembly of the custom-built robotic arm-based multi-axis milling workstation. In this study, a six-axis industrial robotic arm (NB80-80/2.2, ROKAE Robotics Co., Ltd., Jining, China) was employed and fitted with a high-power, electric spindle. This robotic arm has a reach of 2200 mm, and its rated payload is 80 kg. The positional repeatability of this robotic arm is ±0.06 mm, which is suitable for the milling application. The electric spindle was vertically mounted on the sixth joint of the robotic arm (shown in Figure 2b), and its specifications are listed in Table 3. A bull-nose endmill was used for milling (shown as an inset in Figure 2c), and the detailed parameters for this endmill are listed in Table 4.

**Figure 3:**
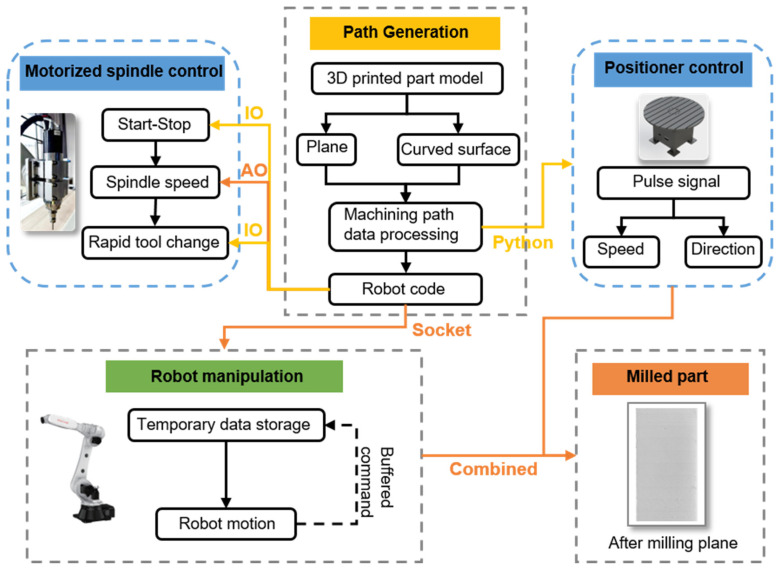
System architecture diagram of the robotic arm-based milling workstation.

The milling experiments in this study were conducted on the robotic arm post-processing workstation, as illustrated in Figure 3. The core workflow includes path planning, code conversion, and real-time control. The Gcode, containing the milling path along with the other critical information, such as feed rate, was first generated by a milling software (such as the machining module in NX (UG 2012, Siemens Digital Industries Software, Plano, TX, USA) or a custom-developed program. Then, a custom-designed Python (Version 3.8) script was employed to translate the Gcode into a format compatible with the robotic arm. For instance, the G01 command (linear interpolation) was mapped to MoveL, and G02/G03 (circular move) to MoveC. In addition, quaternions were subsequently appended to the coordinates, either perpendicular to the path (for non-planar milling) or constant (for planar milling), to ensure the precise orientation control for the endmill.

In addition, a multi-threaded read–write architecture was designed and implemented in the robotic controller to properly regulate the rotational speed of the endmill. More specifically, the robot controller adjusts the spindle speed in real-time (0–24,000 rpm) via the “SetAO” command and controls the spindle’s start/stop and tool changes using the “SetDO” command. The host computer governs the orientation and velocity of the positioning device through the transmission of pulse commands. Concurrently, the primary system disseminates speed and position data in real-time via a specifically designed socket protocol. In order to prevent data loss in serial communication, a circular buffer architecture was designed to pre-store path data and immediately send new instructions after the previous ones are processed, ensuring the continuity of the milling process. A more in-depth description of a similarly designed robotic-arm-based processing platform can be found in an early study [34].

### 2.4. Characterization

**Figure 4:**
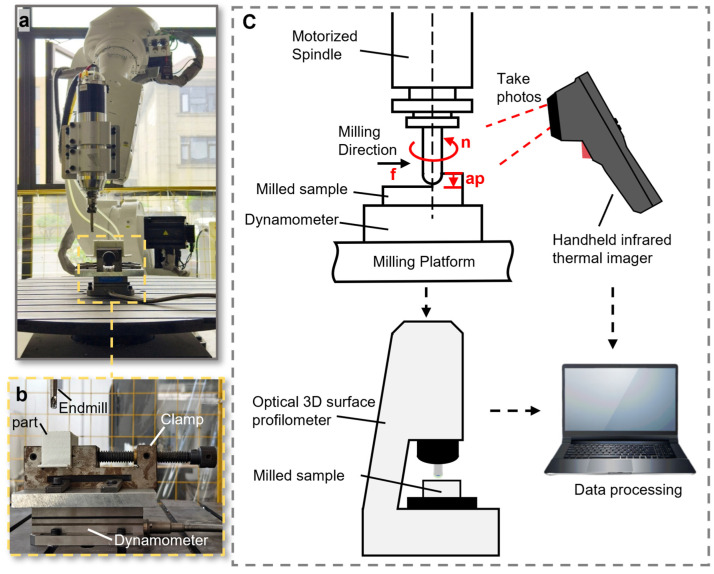
Schematics of the milling experiment: (**a**) picture of the robotic arm post-processing workstation, (**b**) schematics of the force-measurement setup, (**c**) dynamometer, handheld infrared thermal imager, and super W1 optical 3D surface profilometer.

In this experiment, a three-factor, three-level L27 (3^13^) orthogonal design was employed to systematically investigate the effects of process parameters and their interactions on milling performance. This design encompasses 27 parameter combinations. Key responses, including maximum milling temperature, tangential force ($F_{x}$), radial force ($F_{y}$), and surface roughness (Ra), were measured to establish the relationships between parameters and performance. The experiment variables are listed in Table 5. Here, the milling forces were measured using a dynamometer (9257B, Kistler, Winterthur, Switzerland), which was connected to a multi-channel laboratory charge amplifier (5080A, Kistler, Switzerland) and a data acquisition system (5697A, Kistler, Switzerland) (Figure 4c). Milling temperatures were measured using an infrared thermal imager (EO9pro, HIKMICRO, Hangzhou, China) (Figure 4c). Surface roughness was measured using an optical 3D surface profilometer (SuperViewW1, CHOTEST, Shenzhen, China) (Figure 4c). Those measured data were then used in the multi-objective optimization algorithm (NSGA-III, more detailed in the following section), and the optimized milling parameters were ultimately implemented into the machining process to evaluate its effectiveness.

### 2.5. The Multi-Objective Optimization Algorithm

**Figure 5:**
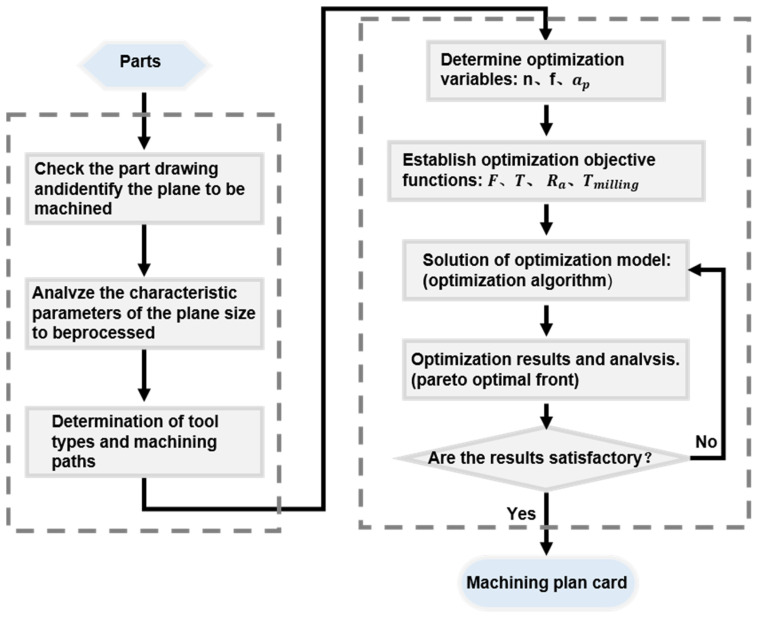
The workflow with the multi-objective optimization process with the NSGA-III algorithm.

Multi-objective optimization algorithms, such as the Non-dominated Sorting Genetic Algorithm III (NSGA-III), are capable of extensively searching and generating multiple solution points within a multidimensional objective space, making them suitable for complex multi-objective optimization scenarios. Here in this study, the NSGA-III algorithm [35] was implemented to efficiently generate a Pareto-optimal set of parameters that balance these objectives, thereby supporting subsequent process validation. The optimization workflow is illustrated in Figure 5.

#### 2.5.1. Establishment of the Optimization Objective Function

Experimental data analysis indicates that spindle speed (*n*), feed rate (*f*), and cutting depth ($a_{p}$) significantly affect milling force, milling temperature, surface quality, and milling time.

(1) Milling force, milling temperature, and surface roughness functions established through polynomial regression models based on experimental parameters

(1) $$Y=\beta_{o}+\sum_{i=3}^{3}\beta_{i}X_{i}+\sum_{i=3}^{3}\beta_{ii}X_{i}^{2}+\sum_{i<j}\beta_{ij}X_{i}X_{j}+\sum_{i=3}^{3}\beta_{iii}X_{i}^{3}+\cdots$$

In this context, the variable $X_{i}$ denotes the independent variable (the process parameter), and the variable β is known as the regression coefficient.

(2) Machining efficiency function based on total time for planar milling

(2) $$T_{milling}=t_{waiting}+t_{air}+t_{cutting}$$

In the given equation, the variable $t_{waiting}$ denotes the standby time, $t_{air}$ is the air cutting time, and $t_{cutting}$ is the material milling time.

#### 2.5.2. Constraints

The constraints of the milling optimization model are summarized as follows:

(3) $$min(F,T,R_{a}{,T}_{milling})=F\left(n,f,a_{p}\right)\;s.t.\left\{\begin{array}{c} n_{min}\leq n\leq n_{max}\\ f_{min}\leq f\leq f_{max}\\ {a_{p}}_{min}\leq a_{p}\leq {a_{p}}_{max}\\ H_{p}=W\cdot a_{p}\\ W_{p}=\left(U-1\right)a_{e}+{∆}_{w} \end{array}\right.$$

where $n_{min}$ and $n_{max}$ are the minimum and maximum spindle speeds allowed during the machining stage, respectively, r/min; $f_{min}$ and $f_{max}$ are the minimum and maximum feed rates allowed during the machining stage, respectively, mm/min; ${a_{p}}_{min}$ and ${a_{p}}_{max}$ are the minimum and maximum milling depths allowed during the machining stage, respectively, mm; $H_{p}$ is the total milling depth, mm; W is the number of milling layers; $W_{p}$ is the total milling width, mm; U is the number of radial milling passes in the machining process; $a_{e}$ is the milling width; ${∆}_{w}$ is the radial machining allowance of the cutting tool in the machining process, mm.

Based on the above analysis of optimization variables, objectives, and constraints, the optimization objectives established in this study are to minimize milling force, milling temperature, surface roughness, and milling time.

## 3. Results and Discussion

### 3.1. Planar Milling

**Figure 6:**
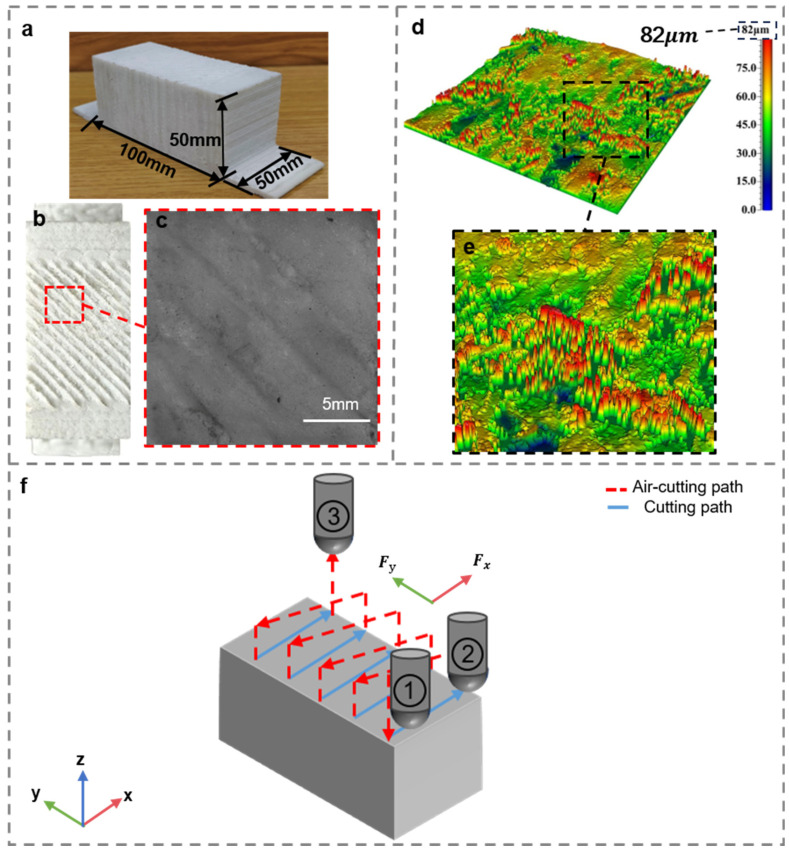
Planar milling process: (**a**) the 3D-printed PP-GF parts, (**b**) the surface of the as-printed part, (**c**) magnified view of the 3D-printed specimen (showing surface waviness), (**d**) surface morphology of the 3D-printed specimen (before milling), (**e**) local magnified view, (**f**) planar milling path planning.

Figure 6a depicts the 3D-printed PP-GF parts, which have dimensions of 50 mm × 100 mm × 50 mm. As can be seen from the magnified image in Figure 6b,c, the surface of the as-printed part exhibits considerable oriented waviness, which aligns well with the infill angle (45°). The surface roughness of this unmilled part is approximately 82 μm (see Figure 6d), accompanied by pronounced sharp protrusions (see Figure 6e). Such a drastically uneven surface makes this part an ideal benchmark for the planar milling process. Figure 6f schematically presents the milling trajectory, which is mainly parallel to the orthogonal X and Y directions. The milling process is carried out sequentially along the trajectory from point 1 to point 2 and then to point 3. By varying three key process parameters, namely spindle speed, feed rate, and milling depth, the milling force (along both the X and Y directions), temperature, and surface roughness were measured separately. The total milling depth was set to 6 mm.

**Figure 7:**
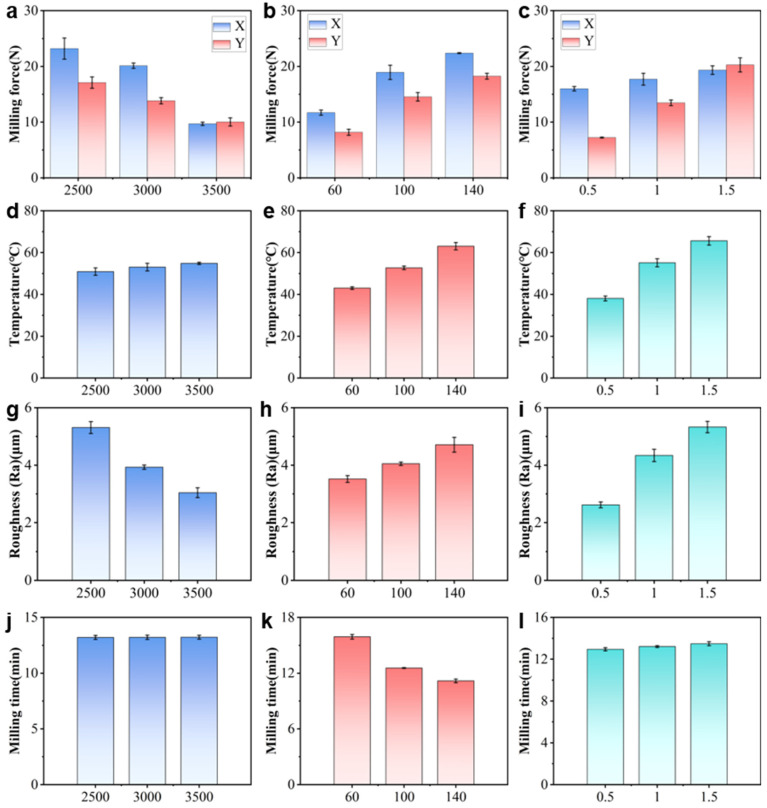
Main effect analysis of factors on milling performance: (**a**) effect of spindle speed on milling force, (**b**) effect of feed rate on milling force, (**c**) effect of cutting depth on milling force, (**d**) effect of spindle speed on milling temperature, (**e**) effect of feed rate on milling temperature, (**f**) effect of cutting depth on milling temperature, (**g**) effect of spindle speed on surface roughness, (**h**) effect of feed rate on surface roughness, (**i**) effect of cutting depth on surface roughness, (**j**) effect of spindle speed on milling time, (**k**) effect of feed rate on milling time, (**l**) effect of cutting depth on milling time.

Figure 7 summarizes the experiment result of the three-factor, three-level L27 (3^13^) orthogonal tests, which highlights the effects of process parameters and their interactions on milling performance. As shown in Figure 7a–c, the milling force is tangible with all three parameters. For instance, an increase in spindle speed decreases the milling force considerably, with the reduction in $F_{x}$ being more pronounced. On the other hand, increasing both the feed rate and cutting depth increases the measured milling force. Such a trend can be explained by the underlying cutting mechanics. A higher spindle speed increases the tool–workpiece interaction frequency and decreases the instantaneous chip thickness, thereby reducing the cutting resistance. In contrast, a higher feed rate enlarges the uncut chip thickness and the material removal rate (MRR), leading to an increased cutting load. These observations are consistent with previous findings reported in the literature [36,37].

As can be seen from Figure 7d–f, the milling temperature slightly increases with the cutting depth and feed rate, yet only modestly increases with the spindle speed. For instance, as the feed rate increases from 60 mm/min to 140 mm/min, the milling temperature rises from approximately 42 °C to 62 °C. This significant increase can be attributed to the higher material removal rate and intensified frictional interaction between the tool and workpiece, which collectively generate more heat and accelerate its transfer to both the tool and the workpiece [33]. Although heat is inevitably generated during the milling process, the overall temperature level remains significantly below the thermal deformation temperature of the PP-GF composite (approximately 110 °C). Within this temperature range, severe thermal effects such as matrix melting, surface smearing, or tool–material adhesion were not observed on the machined surfaces. It is therefore inferred that thermal softening of the polymer matrix is limited under the conditions investigated.

Consequently, milling temperature was treated as a process-monitoring variable rather than an independent optimization objective, ensuring that the selected optimal parameters remain within a thermally safe processing window for PP-GF components, while avoiding redundancy in the multi-objective optimization framework.

As for the surface roughness, it exhibits a similar trend with the milling force, as shown in Figure 7g–i. More specifically, the spindle speed is negatively correlated with surface roughness. In other words, the surface roughness is lower with a higher spindle speed. On the other hand, both increasing the feed rate and the cutting depth are conducive to reducing the surface roughness. For instance, when the milling depth increases from 0.5 mm to 1.5 mm, the surface roughness (Ra) increases from approximately 2.7 μm to 5.5 μm. This deterioration in surface quality can be attributed to the intensified tool vibration and the larger residual cutting area at greater cutting depths, both of which exacerbate the formation of surface irregularities [38].

As illustrated in Figure 7j–l, the feed rate exerts the most pronounced effect on machining efficiency. Increasing the feed rate markedly shortens the machining time as it directly enhances the material removal rate. By comparison, spindle speed and milling depth exert only a minor effect on the total machining time during single-pass milling. Nevertheless, in practical applications, a larger milling depth may lead to additional passes, thereby moderately increasing the overall processing time [39].

To quantify the effects of process parameters, an analysis of variance (ANOVA) was conducted (see Table 6). The results show that the influence of each parameter varies across different response indicators. Milling depth plays a dominant role in surface roughness and milling temperature, while spindle speed and feed rate have more significant effects on the milling force $F_{x}$. Machining time is mainly controlled by the feed rate, whereas the effect of spindle speed is not significant within the tested range. These results provide a quantitative basis for the subsequent multi-objective optimization.

In short, cutting force, cutting temperature, and surface roughness are all significantly influenced by spindle speed, feed rate, and cutting depth. Higher spindle speeds help reduce cutting forces and improve surface quality, while increases in feed rate and cutting depth lead to higher cutting forces and greater surface roughness. Specifically, feed rate has the most significant effect on both cutting force and surface roughness, as higher feed rates increase cutting load and surface roughness. Increasing cutting depth results in higher cutting forces and surface roughness, particularly in terms of cutting temperature and surface quality. These findings provide a theoretical basis for optimizing cutting parameters, contributing to enhanced milling performance.

It should be noted that these conclusions were obtained under controlled experimental conditions. All tests were performed using new or minimally worn tools with relatively short cutting durations, and no evident tool damage or abnormal cutting behavior was observed; therefore, tool wear was not considered a dominant factor in this study. However, its influence is expected to become significant in prolonged machining of glass fiber-reinforced polymers and will be systematically investigated in future work.

### 3.2. Analysis of Planar Milling

The primary objective of milling is to achieve the desired surface quality and dimensional accuracy of the product while maintaining efficient machining. Based on the experimental results, the milling temperature has a relatively minor effect on surface quality. Therefore, the multi-objective optimization focuses primarily on cutting force, surface roughness, and machining time as the core targets.

#### 3.2.1. Approximation with Polynomial Regression

Based on the 27 sets of orthogonal experimental data (Supplementary Information, Table S1), regression models for milling force ($F_{x}$) and surface roughness (Ra) were constructed using the least squares method (see Supplementary Equations (S1) and (S2)). The goodness of fit R^2^ values were 0.971 and 0.978, respectively.

**Figure 8:**
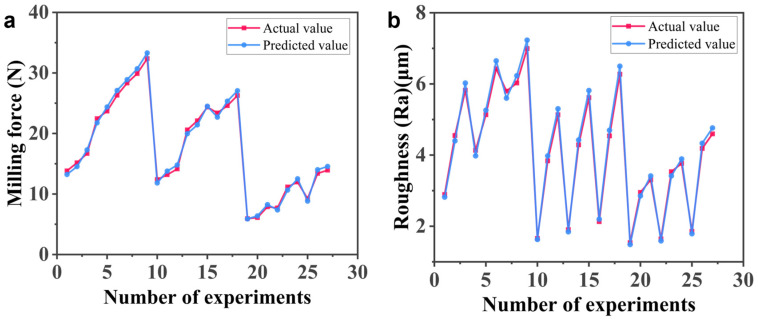
Comparison of experimental and predicted (**a**) milling force and (**b**) surface roughness (Ra).

To further evaluate model robustness and predictive reliability, both validation and residual analyses were conducted. Random validation results showed that the average prediction error was 4.5% for milling force ($F_{x}$) and 3.4% for surface roughness (Ra). A comparison between the predicted and experimentally measured values is presented in Figure 8, demonstrating good agreement without systematic deviation.

In addition, the residual statistical indicators summarized in Table 7 show that the mean residuals for both responses are close to zero, with small standard deviations and limited maximum deviations. These results confirm that the residuals are randomly distributed and that the proposed regression models exhibit good robustness and prediction accuracy within the investigated parameter range, meeting practical engineering requirements.

#### 3.2.2. Efficiency Objective Function

The total milling time ($T_{milling}$) consists of the waiting time, air cutting time, and cutting time: the waiting time $t_{waiting}$ is 5 min (300 s); the air cutting time $t_{air}$ includes time for moving from the origin to the workpiece, lifting the tool, and air cutting paths, which is calculated using Equation (S3) (related to axis coordinates, part dimensions, and air cutting feed speed $V_{air}$; the cutting time $t_{cutting}$ is derived from the volume of material removed and process parameters using Equation (S4). The total time is represented by Equation (S5) (formulas are provided in the Supplementary Information).

#### 3.2.3. Optimization

**Figure 9:**
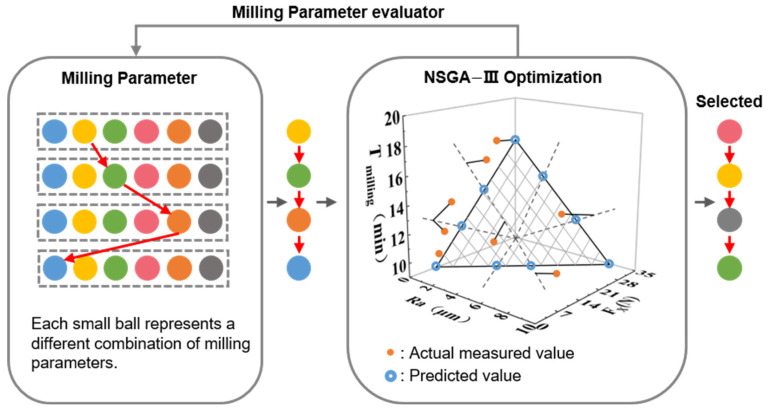
Flow chart of the NSGA III optimization algorithm.

This study adopts the NSGA-III algorithm, implemented in Python, to perform multi-objective optimization of the milling parameters for PP-GF components. Compared with NSGA-II, NSGA-III employs a reference-point–based selection mechanism, which enhances the diversity and uniformity of Pareto-optimal solutions, particularly for problems with conflicting objectives and non-uniform Pareto fronts [40,41]. The specific optimization process of the algorithm is shown in Figure 9, with detailed parameter settings provided in Table 8.

The milling process involves various constraint conditions. In this study, the constraint conditions for face milling are specified in Table 9. These conditions are determined through a comprehensive consideration of the inherent performance parameters of the robotic arm and electric spindle, as well as the recommended cutting parameters for the endmill.

**Figure 10:**
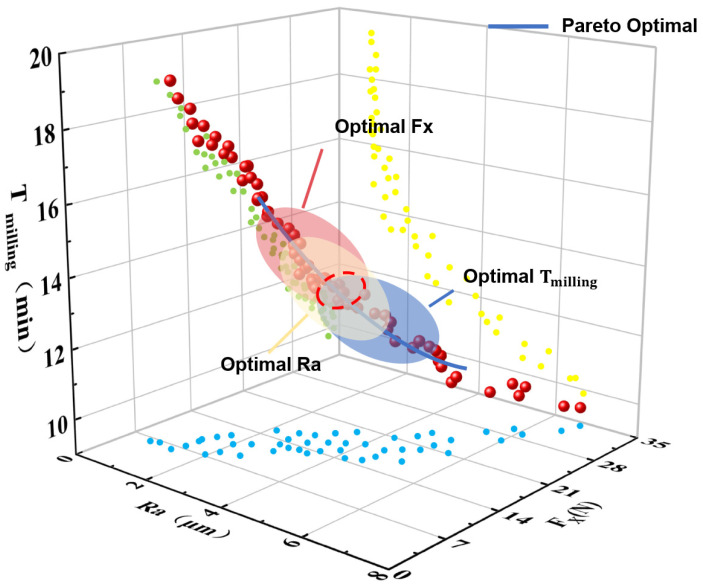
Pareto-optimal fronts for multi-objective optimization of milling performance. The colored dots represent non-dominated solutions under different initial parameter combinations, the colored ellipses indicate the projection of the optimal solution sets for each target (Optimal Fx, Optimal Ra, and Optimal Tmilling), and the red dashed circle marks the final selected compromise optimal solution.

After the parameter configuration was complete, the algorithm was executed iteratively multiple times to optimize the objective function. Using the NSGA-III optimization algorithm, calculations were performed to obtain the Pareto-optimal frontier graph (Figure 10) and output the recommended parameter combinations for practical application scenarios, with specific parameter information listed in Table 10.

**Figure 11:**
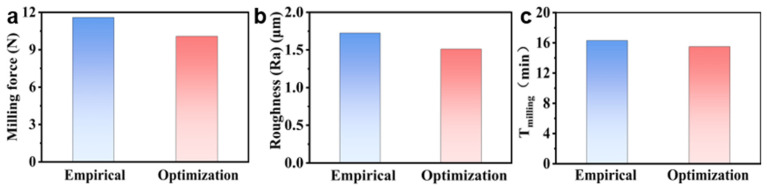
Comparison of target values for the empirical machining scheme and the integrated optimized machining scheme: (**a**) milling force; (**b**) surface roughness; (**c**) milling time.

To verify the practical effectiveness of the proposed optimization strategy, this study selected a set of milling parameters generated by the optimization algorithm and conducted a comparative experiment with milling parameters determined by engineering experience. The face machining allowance was strictly controlled at 0.5 mm during the experiment, which was carried out using the controlled variable method. Figure 11 shows the experimental results, which indicate that the optimized milling parameters could reduce the milling force by 1.51N (a 13.02% decrease), reduce the surface roughness by 0.213 μm (a 12.35% decrease), and shorten the milling time by 0.8 min (a 4.9% decrease) compared with the empirical parameter scheme.

### 3.3. Optimized Planar Milling

**Figure 12:**
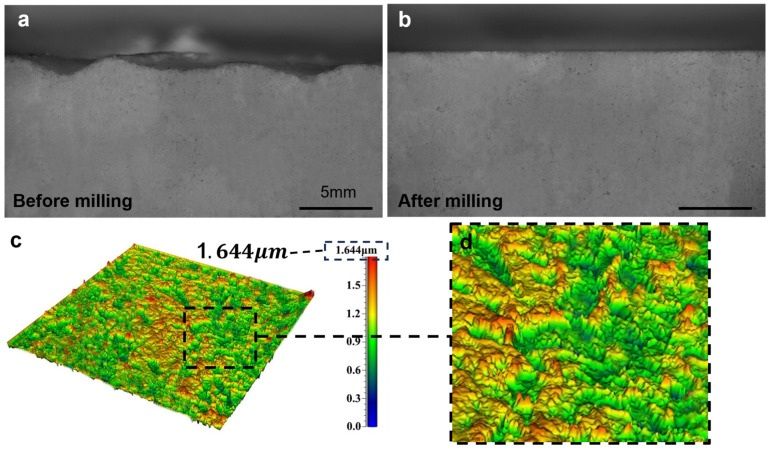
Three-dimensionally printed planar milling specimen: (**a**) cross-sectional view of the printed specimen, (**b**) cross-sectional view after milling, (**c**) surface morphology after milling with spindle speed 3300 r/min, feed rate 70 mm/min, and cutting depth 0.5 mm, (**d**) magnified view of the milled surface.

The milling process was executed using the optimized milling parameters. The wavy morphology of the printed part’s cross-section (Figure 12a) was transformed into a flat profile after milling (Figure 12b), which intuitively demonstrates the improvement effect of milling on surface flatness.

The surface roughness of the milled part decreased to 1.644 μm when the machining parameters were set to a spindle speed of 3300 r/min, a feed rate of 70 mm/min, and a milling depth of 0.5 mm (Figure 12c). Concurrently, the surface microtopography image (Figure 12d) demonstrated that the pronounced protrusions on the surface had been efficiently flattened by milling.

**Figure 13:**
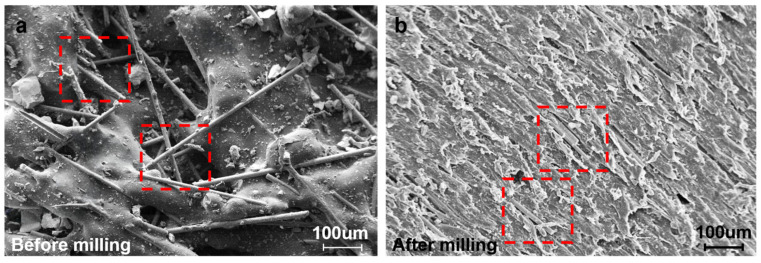
SEM images of PP–GF surfaces: (**a**) as-printed surface with exposed fibers and incomplete fusion; (**b**) surface after robotic milling showing cutting marks and localized fiber fracture and matrix tearing (red dashed boxes).

Figure 13 shows SEM images of PP–GF specimens before and after robotic milling. As shown in Figure 13a, the as-printed surface is characterized by exposed glass fibers, partial fiber pull-out, incompletely fused granules, and irregular surface asperities, resulting in a rough and loosely bonded morphology.

After milling (Figure 13b), the surface becomes noticeably flatter with clear and regular cutting marks. However, localized microstructural damage, including fiber fracture and matrix tearing, can still be observed in the highlighted regions. These features indicate that, although milling significantly improves the macroscopic surface quality, microscale surface integrity is mainly governed by fiber–matrix interfacial failure during machining.

### 3.4. Application of Optimized Parameter for Non-Planar Milling

**Figure 14:**
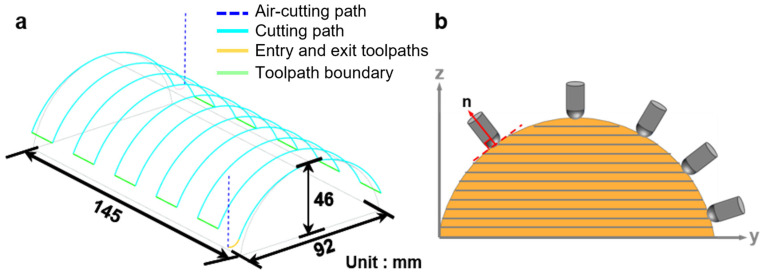
Surface milling toolpath planning and tool orientation constraint: (**a**) surface milling toolpath planning; (**b**) tool axis orientation perpendicular to the machined surface.

In the context of surface milling, the selection of processing methodologies exerts a substantial influence on the stability of the depth of cut and the milling quality. As illustrated in Figure 14, the multi-axis machining mode employing a robotic arm utilizes the multi-axis coordination capabilities to dynamically adjust the tool orientation, thereby ensuring a stable depth of cut and consistent contact with the surface. This outcome yields a level of stability that is analogous to that of planar milling, facilitating the effective transfer of optimization data from planar milling to surface milling. Consequently, this process reduces setup costs and ensures consistent processing quality.

The surface milling path planning is illustrated in Figure 14a, in which a continuous cutting path is generated to ensure stable material removal. To guarantee machining accuracy, the tool axis is required to remain perpendicular to the machined surface, as shown in Figure 14b. This constraint places stringent demands on the posture control and motion coordination of the robotic arm during the milling process.

During the path planning phase, it is imperative that surface normal vector information be extracted in real time and transmitted to the robotic arm control system. By converting the normal vector into a quaternion signal, the robotic arm can dynamically adjust its posture to ensure that the tool remains perpendicular to the surface.

**Figure 15:**
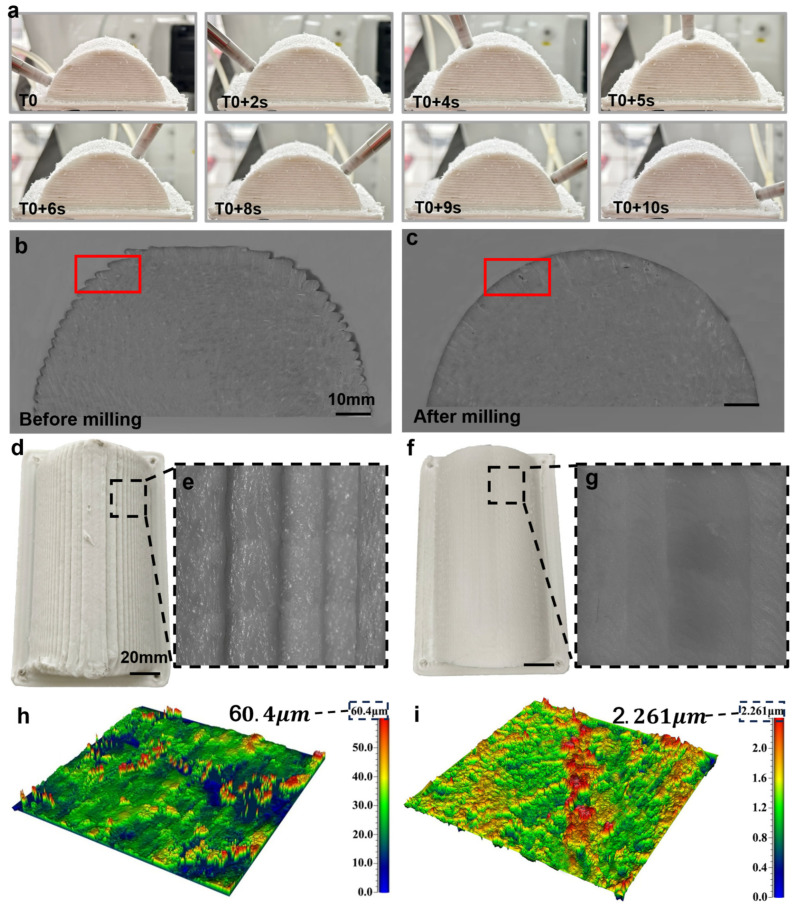
Application of surface milling: (**a**) schematic diagram of the multi-degree-of-freedom milling process, (**b**) cross-sectional view of the printed part, with the red box indicating the local region for surface roughness comparison, (**c**) cross-sectional view of the milled part, with the red box indicating the corresponding local region after milling, (**d**) photograph of the printed part’s surface, (**e**) local magnified view of the printed part’s surface, (**f**) photograph of the milled surface, (**g**) local magnified view of the milled surface, (**h**) surface morphology of the printed part, (**i**) surface morphology after milling.

To leverage the advantages of robotic arm milling in complex surface machining, this study applies the optimized process parameters from planar milling to surface milling and conducts multi-degree-of-freedom surface milling experiments to validate their feasibility and effectiveness. The corresponding experimental results are shown in Figure 15.

As illustrated in Figure 15a, the milling process demonstrates the stability of the robotic arm in executing surface cutting along the predefined trajectory. As illustrated in Figure 15b, high-depth microscopy provides a comprehensive view of the component’s original cross-section. In contrast, Figure 15c showcases the cross-section after milling, a process that effectively eliminates serrated defects and enhances the component’s flatness. With respect to surface quality, Figure 15d presents the overall image of the printed part, while Figure 15e offers a magnified perspective, highlighting substantial surface imperfections, as evidenced by a roughness Ra of 60.4 µm (see Figure 15h). Subsequent to the milling process, it is evident that Figure 15f,g manifest substantial enhancements in surface flatness and smoothness. The surface’s roughness is diminished to 2.261 µm (see Figure 15i), constituting a mere 3.7% of the roughness observed in the printed component.

**Figure 16:**
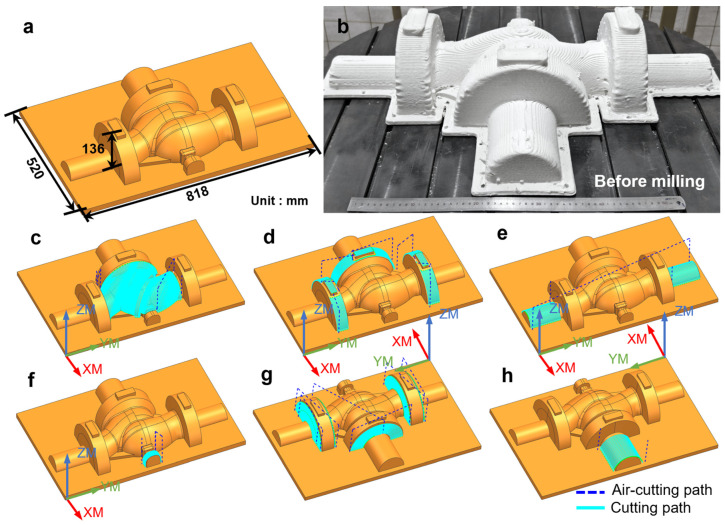
Multi-surface valve model: (**a**) multi-surface valve model, (**b**) 3D-printed valve component, (**c**–**h**) toolpath planning for different surface regions.

Building on the single-surface milling strategy, this work further extends the proposed method to the practical realization of multi-surface milling. As illustrated in Figure 16a, a multi-surface valve model is selected as the representative workpiece, and the corresponding 3D-printed component is shown in Figure 16b. The as-printed surface exhibits pronounced layer-wise textures, along with clearly visible surface irregularities in corner regions. The toolpath planning results for different surface regions are presented in Figure 16c–h, which verify the feasibility and effectiveness of region-based trajectory generation for complex multi-surface machining.

**Figure 17:**
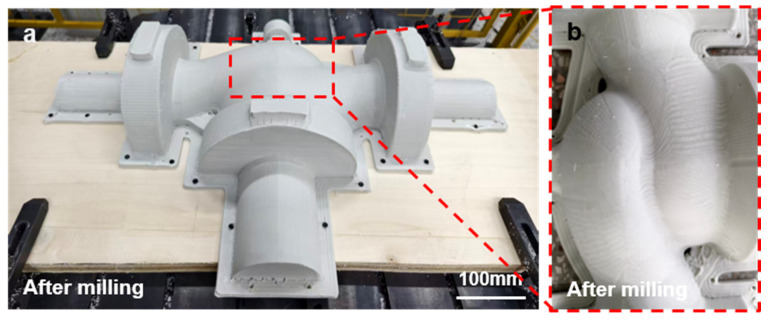
Surface quality of the valve component after robotic milling: (**a**) overall view of the post-milled component, (**b**) local view of the machined central curved surface.

The overall post-milling surface quality is shown in Figure 17a. A magnified view of the machined central complex curved surface is presented in Figure 17b. Compared with the as-printed condition, the milled surface exhibits a substantial improvement in smoothness, with the layer-wise textures being effectively suppressed. As a result, the achieved surface quality satisfies the dimensional accuracy and assembly requirements for industrial applications.

In summary, the parameter transfer and multi-degree-of-freedom processing of the robotic arm effectively improve the surface milling quality, providing a reliable solution for high-precision surface machining.

**Figure 18:**
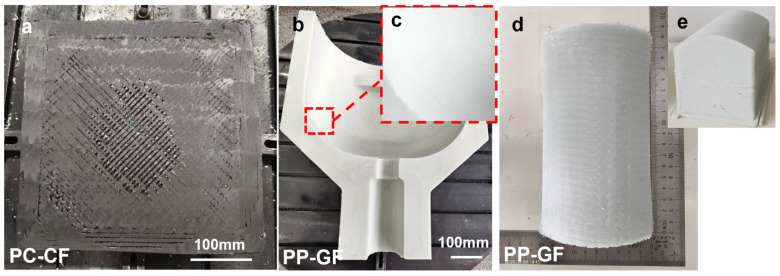
Practical applications of the milling process: (**a**) large-area planar milling of PC-CF parts, (**b**) milling of valve body mold, (**c**) surface finish of curved surface milling, (**d**) milling of semi-cylindrical surfaces, (**e**) side view of the half-cylinder milling.

To further demonstrate the versatility of the proposed approach, Figure 18 presents several additional examples of milled components featuring both planar and non-planar geometries, all processed using the developed robotic arm–based milling platform. Specifically, Figure 18a illustrates the planar milling of 3D-printed parts fabricated from PC–CF material. In Figure 18b,c, the combined machining of planar and curved surfaces on a 3D-printed valve body is successfully achieved, with Figure 18c providing enlarged views of representative regions. Furthermore, the curved-surface milling of 3D-printed half-cylinder components is demonstrated in Figure 18d,e.

## 4. Conclusions

This study focuses on short glass fiber-reinforced polypropylene (PP-GF) material and systematically conducts research on the milling process of large mold components based on fused granular fabrication (FGF)—an extrusion-based 3D printing technology—using a robotic arm post-processing workstation. Through orthogonal experiments, we analyzed the influence of spindle speed, feed rate, and milling depth on milling force, milling temperature, and surface roughness, and clarified the dominant role of each parameter in machining performance. The research results indicate that increasing the spindle speed reduces the milling force and improves the surface quality. However, higher feed rates and milling depths significantly increase the machining heat and roughness. The polynomial regression model constructed based on the experimental data has a high degree of fit (R^2^ > 0.95) and can effectively predict the machining response indicators. Based on this, the NSGA-III multi-objective optimization algorithm was used to determine the optimal milling parameter combination, which reduced the milling force by 13.02%, improved machining efficiency by 4.9%, and decreased surface roughness by 12.35%.

The findings provide valuable theoretical insights and parameter optimization strategies for high-precision machining of 3D-printed, fiber-reinforced composite components, and contribute to the understanding of robotic milling processes applied to FGF-fabricated PP-GF materials. From an engineering perspective, this work offers practical guidance for the application of fused granular fabrication subtractive manufacturing in mold engineering, supporting the deployment of large-scale, 3D-printed molds in precision-demanding industries such as automotive and home appliance manufacturing.

Despite these encouraging results, several limitations should be acknowledged. The milling experiments were conducted over relatively short durations using new cutting tools; moreover, the influence of tool wear was not considered, although it may become significant in long-term machining of glass fiber-reinforced polymer components. In addition, surface integrity evaluation was mainly based on surface roughness and macroscopic morphology. Future research will focus on microscale and subsurface damage characterization, long-term machining stability, and extending the proposed optimization framework to other fiber-reinforced polymer systems and more complex geometric features, thereby further enhancing its industrial applicability.

## Figures and Tables

**Table 1:** Physical and mechanical properties of PP-GF composites.

Fiber Content (wt.%)	Density $\left(\frac{g}{cm^{3}}\right)$	Tensile Strength (MPa)	Bending Strength (MPa)	Deflection Temperature (°C)
30%	1.15	62	65	110 ± 5

**Table 2:** Key printing parameters for the 3D-printing process.

Process Parameters		Values
Infill angle (°)		0–90
Screw temperature (°C)	Heat zone 1	110
	Heat zone 2	170
	Heat zone 3	210
Layer height (mm)		2.0
Line width (mm)		4.5
Printing speed (mm/s)		100

**Table 3:** Parameters for the electric spindle.

Parameters	Value
Rated power	3.5 kW
Maximum rotational speed	24,000 rpm
Dynamic balance grade	G1.0
Cooling method	Water Cooling
Tool changing method	Pneumatic
Weight	15 kg

**Table 4:** Parameters of the bull-nose endmill.

Number of Teeth	Cutting Edge Diameter (mm)	Rotational Speed (rpm)	Tool Shank Diameter (mm)	Total Length (mm)
1	8	2500–3500	12	120

**Table 5:** Factor and level table of the orthogonal experiment.

Level	Spindle Speed/$r\cdot {min}^{-1}$	Feed Rate/$mm\cdot {min}^{-1}$	Milling Depth/mm
1	2500	60	0.5
2	3000	100	1.0
3	3500	140	1.5

**Table 6:** Summary of ANOVA results for different responses.

Response Variable	Spindle Speed (*n*)	Feed Rate (*f*)	Depth of Cut (*a_p_*)	Dominant Factor
Milling Force $F_{x}$	**	**	*	*n*
Milling Temperature	ns	**	**	*a_p_*
Surface Roughness Ra	**	*	**	*a_p_*
Milling time	ns	**	*	*f*

Note: ns—not significant; “*” and “**” indicate significance at *p* < 0.05 and *p* < 0.01, respectively.

**Table 7:** Residual statistical indicators for $F_{x}$ and Ra..

Statistical Indicator	Milling Force $F_{x}$	Surface Roughness Ra
Mean residual	−0.0023 N	0.0015 μm
Standard deviation of residuals	0.876 N	0.187 μm
Maximum positive residual	+1.99 N (Exp27)	+0.423 μm (Exp3)
Maximum negative residual	−1.74 N (Exp7)	−0.398 μm (Exp10)
Sum of squared residuals	20.89	0.924
Coefficient of determination (R^2^)	0.971	0.978

**Table 8:** Parameter setting of NSGA-III.

Parameter	Parameter
Population size	100
Maximum number of generations	200
Evolutionary generation	3
Crossover probability	0.9
Mutation probability	0.1

**Table 9:** Case constraints.

Constraint Conditions	Constraint Range
The range of milling parameters	2500≤n≤3500
	60≤f≤140
	$0\leq a_{p}\leq 1.5$
Constraints on the number of milling layers	$6=W\cdot a_{p}$
Constraint on the number of tool passes	$100=\left(U-1\right)a_{e}+{∆}_{w}$

**Table 10:** Milling parameter table.

Machining Requirements	Spindle Speed/$r\cdot {min}^{-1}$	Feed Rate/$mm\cdot {min}^{-1}$	Milling Depth/mm
High surface quality	3100–3300	60–70	0.5–0.7
High efficiency	3000–3200	100–120	0.6–1.0
Comprehensive optimization	3200–3300	75–85	0.7–0.9

## Data Availability

The raw data supporting the conclusions of this article will be made available by the authors on request.

## Supplementary Materials

The following supporting information can be downloaded at: https://www.mdpi.com/article/10.3390/polym18050608/s1, Table S1: Orthogonal Experimental Design; Table S2: Analysis of Variance (ANOVA) for Milling Force Fx; Table S3: Analysis of Variance (ANOVA) for Milling Temperature; Table S4: Analysis of Variance (ANOVA) for Surface Roughness (Ra); Table S5: Analysis of Variance (ANOVA) for Milling Time.
