# Supplementary material for: Six-Axis Robotic Milling for Enhancing Surface Quality and Dimensional Accuracy of Fused Granular Fabrication Parts
Source: Polymers (Basel). 2026 Feb 28;18(5):608. doi: 10.3390/polym18050608 (PMC12987160; doi:10.3390/polym18050608)
Supplement: Supplementary file 1 [file polymers-18-00608-s001.zip › polymers-4129510-supplementary.pdf]

Table S1. Orthogonal Experimental Design

Experimental group	Spindle Speed $/ r \cdot min^{-1}$	Feed Rate /mm/s	Depth of Cut/ mm	Milling Temperature/℃	Milling Force F_x/N	Milling Force F_y/N	Surface Roughness Ra/μm	Milling time/min
Exp1	2500	60	0.5	29.3	13.83	7.44	2.891	15.5
Exp2	2500	60	1	43.1	15.17	9.64	4.552	15.9
Exp3	2500	60	1.5	52.7	16.69	13.00	5.821	16.2
Exp4	2500	100	0.5	33.1	22.46	11.13	4.136	12.4
Exp5	2500	100	1	52.5	23.69	17.73	5.136	12.5
Exp6	2500	100	1.5	66.3	26.32	25.99	6.430	12.8
Exp7	2500	140	0.5	50.3	28.33	14.08	5.798	10.9
Exp8	2500	140	1	62.3	29.89	21.65	6.027	11.1
Exp9	2500	140	1.5	68.5	32.37	33.18	6.994	11.5
Exp10	3000	60	0.5	29.3	12.39	4.57	1.658	15.8
Exp11	3000	60	1	45.9	13.19	8.52	3.838	15.9
Exp12	3000	60	1.5	54.5	14.14	11.47	5.133	16.1
Exp13	3000	100	0.5	33.2	20.60	7.79	1.905	12.3
Exp14	3000	100	1	55.3	22.11	14.18	4.288	12.5
Exp15	3000	100	1.5	68.8	24.36	22.18	5.614	12.9
Exp16	3000	140	0.5	52.2	23.40	7.87	2.132	10.7
Exp17	3000	140	1	63.9	24.60	18.43	4.539	11.2
Exp18	3000	140	1.5	74.3	26.27	29.50	6.273	11.5
Exp19	3500	60	0.5	30.0	5.94	3.16	1.538	15.8
Exp20	3500	60	1	46.7	6.10	6.78	2.952	15.9
Exp21	3500	60	1.5	55.7	7.89	9.12	3.306	16.1
Exp22	3500	100	0.5	32.5	7.71	4.14	1.644	12.2
Exp23	3500	100	1	58.4	11.17	10.70	3.534	12.6
Exp24	3500	100	1.5	74.3	11.98	17.01	3.768	12.8
Exp25	3500	140	0.5	52.7	9.25	4.92	1.857	10.9
Exp26	3500	140	1	68.0	13.40	13.54	4.188	11.3

Exp27	3500	140	1.5	75.3	13.93	20.98	4.597	11.4

Table S2. Analysis of Variance (ANOVA) for Milling Force Fx

**Factor**	**Sum of Squares (SS)**	**Degrees of Freedom (df)**	**Mean Square (MS)**	**F-value**	**Significance**	**Contribution (%)**
Spindle speed (n)	899.17	2	449.59	107.64	**	**57.37**
Feed rate (f)	534.5	2	267.25	63.99	**	**34.1**
Depth of cut (a_p)	50.15	2	25.07	6	*	3.2
Error	83.53	20	4.18	—	—	5.33
Total	1567.35	26	—	—	—	100

Table S3. Analysis of Variance (ANOVA) for Milling Temperature

**Factor**	**Sum of Squares (SS)**	**Degrees of Freedom (df)**	**Mean Square (MS)**	**F-value**	**Significance**	**Contribution (%)**
Spindle speed (n)	70.19	2	35.1	2.81	ns	1.25
Feed rate (f)	1806.65	2	903.33	72.37	**	32.25
Depth of cut (a_p)	3476.28	2	1738.14	139.26	**	62.05
Error	249.63	20	12.48	—	—	4.45
Total	5602.76	26	—	—	—	100

Table S4. Analysis of Variance (ANOVA) for Surface Roughness (Ra)

**Factor**	**Sum of Squares**	**Degrees of**	**Mean Square**	**F-value**	**Significanc**	**Contribution**

	(SS)	Freedom (df)	(MS)		e	(%)
Spindle speed (n)	23.48	2	11.74	41.31	**	33.84
Feed rate (f)	6.41	2	3.2	11.27	*	9.23
Depth of cut (a_p)	33.82	2	16.91	59.5	**	48.74
Error	5.68	20	0.28	—	—	8.19
Total	69.4	26	—	—	—	100

Table S5. Analysis of Variance (ANOVA) for Milling Time

**Factor**	**Sum of Squares (SS)**	**Degrees of Freedom (df)**	**Mean Square (MS)**	**F-value**	**Significance**	**Contribution (%)**
Spindle speed (n)	0.52	2	0.26	1.92	ns	0
Feed rate (f)	108.73	2	54.37	402.01	**	98.64
Depth of cut (a_p)	2.41	2	1.21	8.94	*	1.18
Error	2.71	20	0.136	—	—	0.18
Total	114.37	26	—	—	—	100

$$F_x = -47.21 - 0.0278 \cdot \mathrm{n} - 2.1 \times 10^{-5} \cdot \mathrm{n}^2 + 0.0083 \cdot \mathrm{f} + 1.22 \times 10^{-3} \cdot \mathrm{f}^2 + 5.61 \cdot a_p + 6.388 \cdot {a_p}^2 + 0.149 \cdot \mathrm{f}a_p + 3.736 \cdot {a_p}^3 - 3.49 \times 10^{-4} \cdot \mathrm{f} \cdot {a_p}^2 \quad \text{(S1)}$$

$$Ra = -32.54 + 0.0123 \cdot \mathrm{n} - 2.1 \times 10^{-5} \cdot \mathrm{n}^2 + 0.051 \cdot \mathrm{f} + 5.82 \cdot a_p - 3.2 \times 10^{-4} \cdot \mathrm{f}^2 + 8.7 \times 10^{-6} \cdot \mathrm{f}^2 a_p - 0.0603 \cdot {a_p}^2 - 1.1 \cdot \mathrm{n}^2 a_p \quad \text{(S2)}$$

$$t_{air} = t_z + t_{up} + t_n = \left(\frac{2 \times L_z}{V_{air}} + \frac{2 \times \left(\frac{L_{part}}{a_e} - 1\right) \times 50}{V_{air}} + \frac{\left(\frac{L_{part}}{a_e} - 1\right) \times W_{part}}{V_{air}} \right) \times 60 = 60 \times \left(\frac{2 \times 100 + \left(\frac{100}{a_e} - 1\right) \times 150}{V_{air}} \right) \tag{S3}$$

Here, t_z represents the time from the reference point to the workpiece, t_{up} is the tool lift time, and t_n denotes the idle milling (air-cutting) time. L_x、L_y、L_z are the relative coordinates of the reference point to the workpiece along the X, Y, and Z axes, respectively. L_{part} and W_{part} correspond to the length and width of the workpiece to be machined, and $Vair$ represents the feed rate during idle (air-cutting) movement.

$$t_{cutting} = 60 \times \frac{V_r}{f \times a_p \times a_e} = 60 \times \frac{50 \times 100 \times a_p}{f \times a_p \times a_e} \tag{S4}$$

Here, V_r represents the volume of material removed.

$$T_{milling} = 60 \times \left(\frac{2 \times 100 + \left(\frac{100}{a_e} - 1\right) \times 150}{V_{air}} + \frac{50 \times 100 \times a_p}{f \times a_p \times a_e} \right) + 300 \tag{S5}$$
